# Optimisation of Cellulase Production by *Penicillium funiculosum* in a Stirred Tank Bioreactor Using Multivariate Response Surface Analysis

**DOI:** 10.1155/2014/703291

**Published:** 2014-06-25

**Authors:** Marcelle Lins de Albuquerque de Carvalho, Daniele Fernandes Carvalho, Edelvio de Barros Gomes, Roberto Nobuyuki Maeda, Lidia Maria Melo Santa Anna, Aline Machado de Castro, Nei Pereira

**Affiliations:** ^1^Bioprocess Development Laboratory, Biochemical Engineering Department, School of Chemistry, Federal University of Rio de Janeiro, P.O. Box 68542, 21945-970 Rio de Janeiro, RJ, Brazil; ^2^Biotechnology Division, Research and Development Center, PETROBRAS, Avenida Horácio Macedo, 950 Ilha do Fundão, 21941-915 Rio de Janeiro, RJ, Brazil

## Abstract

Increasing interest in the production of second-generation ethanol necessitates the low-cost production of enzymes from the cellulolytic complex (endoglucanases, exoglucanases, and *β*-glucosidases), which act synergistically in cellulose breakdown. The present work aimed to optimise a bioprocess to produce these biocatalysts from the fungus *Penicillium funiculosum* ATCC11797. A statistical full factorial design (FFD) was employed to determine the optimal conditions for cellulase production. The optimal composition of culture media using Avicel (10 g*·*L^−1^) as carbon source was determined to include urea (1.2 g*·*L^−1^), yeast extract (1.0 g*·*L^−1^), KH_2_PO_4_ (6.0 g*·*L^−1^), and MgSO_4_ *·*7H_2_O (1.2 g*·*L^−1^). The growth process was performed in batches in a bioreactor. Using a different FFD strategy, the optimised bioreactor operational conditions of an agitation speed of 220 rpm and aeration rate of 0.6 vvm allowed the obtainment of an enzyme pool with activities of 508 U*·*L^−1^ for FPase, 9,204 U*·*L^−1^ for endoglucanase, and 2,395 U*·*L^−1^ for *β*-glucosidase. The sequential optimisation strategy was effective and afforded increased cellulase production in the order from 3.6 to 9.5 times higher than production using nonoptimised conditions.

## 1. Introduction

The conversion of lignocellulosic materials into bioethanol has gained extensive attention in recent years due to the increasing scarcity of fossil fuels and growing interest in the domestic production of biofuels [1]. Environmental issues, such as the reduction of carbon dioxide emission by blending bioethanol with gasoline, have increased the interest in production of biofuel from lignocellulose. Enzymatic hydrolysis to convert cellulose into fermentable sugars has been studied extensively because this is one of the crucial steps of bioethanol production [2], presenting high significance on the economic aspects of this process [3]. There is a general interest in obtaining cellulase enzymes that are more specific and stable [4], preferably in an on-site configuration, in order to reduce logistic costs [3]. In the bioethanol production process, enzymatic hydrolysis can occur separately or sequentially to fermentation of the released sugars [5].

There are at least three categories of enzymes that convert cellulose (a linear, typically insoluble polymer containing thousands of glucose units) into soluble sugars [6]. The cellulolytic complex includes the following: endoglucanase (EG, EC 3.2.1.4), which randomly catalyzes the hydrolysis internal *β*-1,4 glycosidic bonds in the cellulose chain; cellobiohydrolase (CBH, EC 3.2.1.91), which moves progressively along the cellulose chain and catalyzes the release of cellobiose units from the chain's terminus; and *β*-glucosidase (BG, EC 3.2.1.21), which converts cellobiose and soluble cellodextrins into glucose. All of these enzymes act synergistically to hydrolyse cellulose through the creation of new accessible sites for each other and the prevention of product inhibition [7]. In the production of cellulosic ethanol, the most common application of cellulases is as a whole broth (i.e., the whole fermentation broth from enzyme production step, with cells) and as a culture filtrate (either concentrated or not) [8, 9].

The majority of cellulose degradation occurs through fungal or bacterial attack [10]. Some of the fungi used for industrial production of cellulases are from the genera* Trichoderma*,* Humicola*,* Aspergillus,* and* Penicillium* [11–13]. Over the last 50 years, one of the most studied fungi has been* T. reesei*, which is known to produce at least two exoglucanases, five endoglucanases, and two *β*-glucosidases [14, 15]. One of the main limitations of the* T. reesei* cellulolytic system is the low production of *β*-glucosidase compared to other groups of cellulases [16]. On the other hand,* Aspergillus* species are known to be excellent *β*-glucosidase producers but have relatively low endoglucanase production. For these reasons, several studies have focused on coculture of fungi from both genera to produce equivalent amounts of cellulolytic complex enzymes [15, 17, 18].

*Penicillium* strains have been reported as producers of cellulolytic complexes with improved synergy due to their high production of *β*-glucosidase and endoglucanase [19]. In particular,* P. funiculosum* ATCC 11797 has recently been identified as an outstanding source of well-balanced cellulolytic complexes [20]. When used in the form of its enzyme extract, either alone or blended with* T. harzianum* cellulolytic extract, it has been shown to have great potential for biomass hydrolysis, yielding up to 86% cellulose conversion [9]. Although* P. funiculosum* can efficiently produce cellulases from pretreated agroindustrial residues [20, 21], there is a great variability in the source and composition of such materials that negatively affects the reproducibility of results obtained under optimised conditions. The present study investigated the optimisation of culture conditions for cellulase production by* Penicillium funiculosum* ATCC 11797. This organism was grown by submerged fermentation using certified model carbon sources as substrates, and the culture medium composition and operational conditions were modified with the aim of maximising the rate of enzyme production.

## 2. Material and Methods 

### 2.1. Microorganism Growth and Maintenance

*P. funiculosum *ATCC 11797 was obtained from the Instituto Oswaldo Cruz (FIOCRUZ, Rio de Janeiro, RJ, Brazil) culture collection. The strain was maintained on PDA (potato, dextrose, agar) plates (DIFCO, Franklin Lakes, NJ, USA) at 30°C for 9-10 days before inoculation.

### 2.2. Production of Cellulase from Certified Model Carbon Sources

Resuspended spores of* P. funiculosum *(5.33 × 10^7^, total amount) were used to inoculate 100 mL of modified Mandels and Weber medium [22] in 500 mL conic flasks, which were incubated at 30°C and 200 rpm for cell propagation. After 3 days, 10 mL of culture containing the growing cells (3.8 g·L^−1^) was transferred into 1 L Erlenmeyer flasks containing 200 mL of media containing supplemented with Avicel CE-15 (10 g/L, microcrystalline cellulose, FMCBiopolymer, Philadelphia, USA), medium viscosity carboxymethylcellulose (CMC), or cellobiose (Sigma, St Louis, MO, USA). Cultures were incubated at 200 rpm and 30°C for 192 hours. Samples were collected at regular intervals and centrifuged at 20,000 ×g for 5 min to harvest cells and residual substrate [20]. Supernatants were removed, frozen, and stored for later assay. Data were analysed using the software Statistica 6.0 (Statsoft Inc., Tulsa, OK, USA).

### 2.3. Experimental Design for Optimisation of Culture Medium

A full factorial design (2^4^ FFD) was carried out to optimise the culture media composition (Table 1). The nutrients used for this optimisation were selected based on results from a previous screening using factorial design (data not shown). The conditions for inoculum propagation and sample treatment were the same as described in Section 2.2.

### 2.4. Optimisation of Bioreactor Operational Conditions

To determine the best operational conditions for cellulase production, 1 L of optimised culture medium was inoculated in a 2 L stirred tank bioreactor (Biostat B, B. Braun Biotech International, Allentown, USA). The pH of the medium was maintained at 5.0 via addition of NaOH (2 M) or HCl (2 M), and the temperature was maintained at 30°C. Agitation speed and aeration rate were considered to be the most important variables and used for a full factorial design (3^2^ FFD). The levels and factors considered in this FFD are presented in Table 2. The conditions for inoculum propagation and sample treatment were the same as described in Section 2.2.

### 2.5. Assays

Activities were determined for FPase (filter paper degradation), endoglucanase, and *β*-glucosidase using Whatman number 1 filter paper, medium viscosity CMC, and cellobiose as substrates, respectively, using slight modifications of previously described conditions [23]. These protocols were set as standards for subsequent analyses. Glucose obtained from the *β*-glucosidase reaction was quantified using an analytical kit utilising glucose oxidase and peroxidase for detection (Laborlab, São Paulo, SP, Brazil). Total extracellular protein content was measured using the Bio-Rad protein reagent (Bio-Rad Laboratories, Hercules, CA, USA) according to the Bradford method [24] with bovine serum albumin (BSA) (Sigma, St. Louis, MO, USA) as a standard. All analyses were performed in triplicate in a temperature-controlled incubator (Dubnoff, Nova Técnica, São Paulo, SP, Brazil).

## 3. Results and Discussion

### 3.1. Evaluation of Model Carbon Sources for Cellulase Production

Three model cellulosic substrates were evaluated to investigate their potential as carbon sources for cellulase production by* P. funiculosum*. Fermentation kinetics was monitored over periods of up to 96 hours. As shown in Table 3, Avicel promoted the highest cellulase production as indicated by the maximum activity observed during fermentation for the three activities evaluated. Avicel was used for all subsequent optimisation steps.

### 3.2. Culture Medium Optimisation through Experimental Design

The first experimental design was applied in order to determine the most appropriate source and concentration of nutrients, which were selected based on a previous experimental design in which a larger set of nutrients was tested (data not shown) [25]. In these experiments, cellulase activities and protein concentrations were determined after 120 hours of fermentation in conical flasks. The results from the 2^4^ FFD are presented in Table 4. The highest cellulase activity and protein concentrations were observed using the central point conditions. FPase, endoglucanase, and *β*-glucosidase activities reached values of 168 ± 8, 5078 ± 431, and 1271 ± 90 U·L^−1^, respectively.

**Figure 1:**
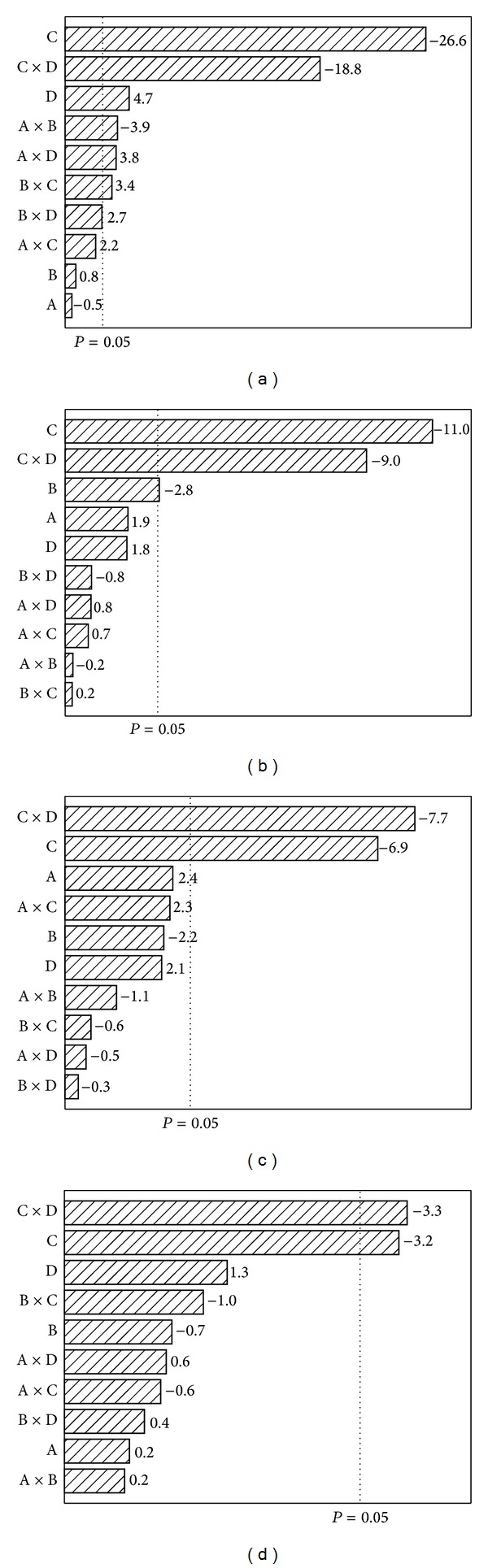
Pareto chart showing effect of nutrients on the following: (a) FPase activity, (b) endoglucanase activity, (c) *β*-glucosidase activity, and (d) protein concentration. A, B, C, D: see Table 1.

This 2^4^ FFD allowed detection of the statistically significant concentrations of nutrients required for cellulase production by* P. funiculosum* ATCC 11797 using Avicel as the main carbon source.  Figure 1 shows the Pareto charts for the three enzyme activities and the protein concentration. Dotted vertical lines represent the limit between statistically significant and nonsignificant factors (single or interactions) using a 95% confidence interval (*P*-level = 0.05).

Based on statistical analysis of the results, the composition of the medium selected for use in further studies contained urea (1.2 g·L^−1^), yeast extract (1.0 g·L^−1^), KH_2_PO_4_ (6.0 g·L^−1^), and MgSO_4_·7H_2_O (1.2 g·L^−1^).

In the past years, several species of* Penicillium* that produce cellulolytic enzymes have been reported. A study published by Krogh et al. [26] investigated the production of cellulases in conical flasks by twelve strains of the* Penicillium* genus. Using Solka-Floc as substrate, the maximum *β*-glucosidase activity was 2450 U·L^−1^ (*P. pinophilum* IBT 10872), and the highest FPase activity was 680 U·L^−1^ (*P. brasilianum* IBT 20888) [26]. The fungus* P. echinulatum* was studied by Martins et al. [27], who found the maximum FPase, endoglucanase, and *β*-glucosidase activities after 192 hours of growth to be 270, 1530, and 190 U·L^−1^, respectively. Adsul et al. [28] described production of these enzymes by* P. janthinellum* NCIM 1171 and reported maximum FPase and *β*-glucosidase activities of 1500 and 7000 U·L^−1^, respectively. Finally, Jørgensen et al. [19] evaluated the production of cellulases in bioreactor by three* Penicillium* strains using the model substrate Solka-Floc as the source of cellulose; the maximum FPase activity observed by culturing* P. brasilianum* IBT 20888,* P. pinophilum* IBT 4186, and* P. persicinum* IBT 13226 was 750 U·L^−1^ (229 hours), 280 U·L^−1^ (221 hours), and 800 (236 hours) U·L^−1^, respectively.

### 3.3. Operational Conditions Optimisation in Tank Bioreactor

Working with an instrumented bioreactor, the effects of agitation speed and aeration rate were simultaneously studied using a 3^2^ FFD. These are considered the most critical variables in the production of cellulase enzymes in bioreactor systems because the influence of other variables, such as pH (either initial pH or pH throughout the fermentation, in case of use of a suitable buffer) and temperature, is feasible to be alternatively determined in simpler systems such as shaking flasks. The experimental results, observed after 120 hours of fermentation using parameters established from the 3^2^ FFD, are presented in Table 5.

The results were analysed using statistical approaches to investigate linear, quadratic, and interaction effects. Then, the results were fitted to second-order models, and the regression coefficients were combined into equations to determine the response values for the production of enzymes (1)–(3) as well as total extracellular protein (4), as a function of agitation (*E*) and aeration (*F*). Only the statistically significant terms (using a 95% confidence interval) that were validated through analysis of variance (ANOVA) are shown in the following equations:

(1) FPase  activity (U·L−1)=(−0.732+0.009∗E+0.461∗F−0.253∗F2)∗1000

(2) Endoglucanase  activity (U·L−1)=(−11.133+0.105∗E+25.840∗F −24.134∗F2+0.014∗E∗F)∗1000

(3) β-glucosidase  activity (U·L−1)=(−1.477+0.005∗E+8.308∗F−6.947∗F2)∗1000

(4) Protein  concentration (mg·L−1)=(−0.233+0.003∗E+0.092∗F)∗1000.

Partial derivation of (1)–(4) reveals critical conditions that maximise or minimise the response values. These conditions and the corresponding values observed experimentally are shown in Table 6. The four maximum and minimum response values were not optimally obtained using the same agitation and aeration conditions. For this reason, a multivariate analysis was adopted using a global desirability function (*D*_*f*_) to achieve maximisation of all four response variables together.

This function converts each response (*y*_*i*_) into an individual desirability function (*d*_*i*_) varying in a range from 0 to 1 (0 ≤ *d*_*i*_ ≤ 1). This function, shown in (5), allows determination of values for each independent variable to maximise *D*_*f*_ [29]. Consider

(5) $$\begin{array}{c} D_{f}={\left(d_{1}*d_{2}*d_{3}*d_{4}\right)}^{0.25}. \end{array}$$

**Figure 2:**
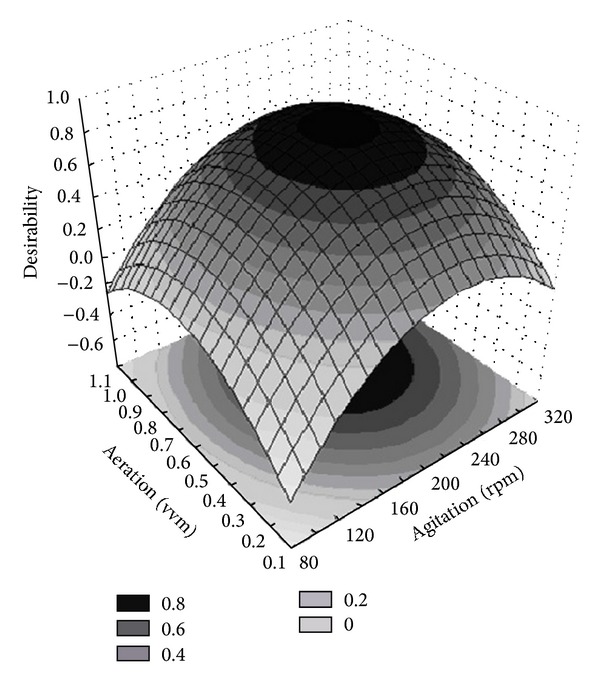
The response surface for the desirability function of 3^2^ FFD analysis used for optimisation of cellulase production.

The surface response for the desirability function is presented in Figure 2. The optimum operational conditions predicted by this multivariate analysis are 227.5 rpm for agitation speed and 0.68 vvm for aeration rate, with a corresponding global desirability value *D*_*f*_ = 0.83.

### 3.4. Validation of the Optimal Conditions Predicted by Multivariate Analysis

**Figure 3:**
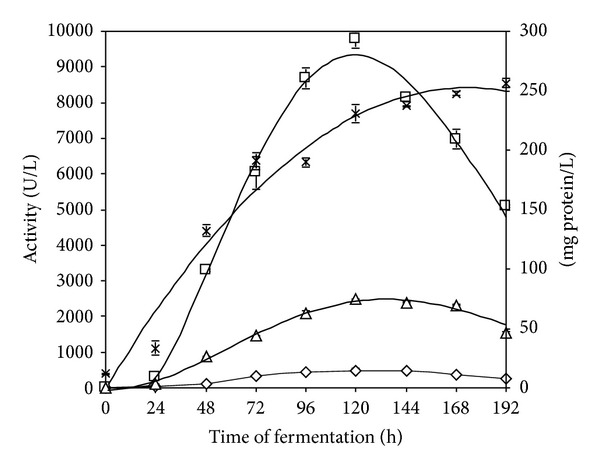
Cellulase production using an instrumented bioreactor at optimal conditions (optimal medium, 220 rpm and 0.6 vvm). (-◊-) FPase activity; (-□-) endoglucanase activity; (-Δ-) *β*-glucosidase activity; and (-x-) protein concentration.

To validate the results obtained by the application of the desirability function to the 3^2^ FFD data, independent experiments were performed using the combination of aeration rate and agitation speed values predicted to provide the best conditions for maximising enzyme activity and protein concentration. The individual and global predicted conditions that maximise D_f_ are presented in Table 6 along with the experimental values observed in the bioreactor experiments. Production of cellulases by* P. funiculosum* in an instrumented bioreactor using the variables fit very closely with the optimal values pointed by the model, resulting in equivalent or higher enzyme activities than those predicted by statistical analysis. The highest cellulase production observed experimentally corresponds to an agitation speed of 220 rpm and an aeration rate of 0.60 vvm.  Figure 3 shows cellulase activities and protein concentrations using these conditions. The maximum volumetric productivities for FPase, endoglucanase, and *β*-glucosidase enzymes were 4.6 U·(L·h)^−1^ (72 hours), 95 U·(L·h)^−1^ (96 hours), and 21 U·(L·h)^−1^ (96 hours), respectively.

Jatinder et al. [30] studied the optimisation of culture conditions for cellulase production by* Humicola Insolens* MTCC 4520 using rice straw and wheat bran (1 : 3 m/m) as a carbon source. Through solid-state fermentation using response surface methodology, FPase, endoglucanase, and *β*-glucosidase with respective activities of 3.0, 62.5, and 151 U·(g substrate)^−1^, respectively, were produced [24]. Alam et al. [31] used a fractional factorial design with six factors to determine the optimal processing conditions for cellulase production by* Trichoderma harzianum* from domestic wastewater sludge; statistical analysis and surface response show the maximum production of filter paper hydrolysing enzymes to be 10,200 U·L^−1^ after three days of fermentation [31].

According to Castro et al. [20] the values of filter paper, endoglucanase, and *β*-glucosidase activities were 250 U·L^−1^, 1800 U·L^−1^, and 800 U·L^−1^, respectively, using the same strain of* P. funiculosum* as the utilised in the present work, with sugarcane bagasse cellulignin as carbon source and a 120-hour growth period. In this work, Avicel proved to be an interesting model substrate for cellulase production, yielding higher activities when compared to previously reported cellulosic substrates [20]. Fermentation of* P. funiculosum *ATCC 11797 in Avicel showed productivity values 5 to 33 times higher than those observed using strains from different genera, such as* T. reesei* Rut C30 and* Humicola grisea* and other strains of* Penicillium* genus [32]. Through the process optimisation strategies adopted, the FPase, endoglucanase, and *β*-glucosidase activities were increased by 9.5 times compared with values observed prior to optimisation. The sequential experimental design strategy was therefore effective for optimisation of cellulase production by* P. funiculosum*. Finally, since the variables evaluated in this study (aeration and stirring speed) may be influenced by the power number, it should be stressed that for future scale up of this process, this parameter such as *k*_*L*_ a should be considered [33].

## 4. Conclusion

*Penicillium funiculosum* ATCC 11797 can efficiently produce cellulolytic enzymes utilising Avicel as the sole carbon source using a submerged fermentation process. Optimal processing conditions determined using multivariate statistical analysis yielded the maximum activities for FPase, endoglucanase, and *β*-glucosidase to be 508, 9204, and 2395 U·L^−1^, respectively. Full factorial design and multivariate response surface analyses were successfully applied to optimise the fermentation and growth process, allowing a 9.5-fold increase in productivity.

## Figures and Tables

**Table 1:** Levels of 2^4^ FFD for culture medium optimisation.

Variable	Low level	Center point	High level
*A*-KH_2_PO_4_ (g*·*L^−1^)	4.0	6.0	8.0
*B*-MgSO_4_ *·*7H_2_O (g*·*L^−1^)	0.6	1.2	1.8
*C*-Urea (g*·*L^−1^)	0.6	1.2	1.8
*D*-Yeast extract (g*·*L^−1^)	0.5	1.0	1.5

**Table 2:** Levels of 3^2^ FFD for optimisation of process conditions in bioreactor.

Variable	Low level	Center point	High level
*E*-Agitation (rpm)	100	200	300
*F*-Aeration rate (vvm)	0.2	0.6	1.0

**Table 3:** Maximum cellulase activities in different model cellulosic substrates.

Substrate	Highest activity (U*·*L^−1^)^a^		
	FPase	Endoglucanase	*β*-Glucosidase
Avicel	86.6 ± 3.1 (72)	590.1 ± 26.3 (72)	168.4 ± 15.8 (72)
Cellobiose	14.7 ± 4.0 (24)	72.7 ± 6.3 (24)	43.4 ± 0.3 (48)
CMC	37.6 ± 2.1 (72)	309.3 ± 20.0 (72)	83.3 ± 7.5 (96)

^a^Values in parentheses correspond to time of fermentation (h) when the maximum activities were observed.

**Table 4:** Cellulase activities and protein concentration for the 2^4^ FFD for medium optimisation.

Run	*A* ^ a^	*B* ^ a^	*C* ^ a^	*D* ^ a^	FPase activity (U*·*L^−1^)	Endoglucanase activity (U*·*L^−1^)	*β*-Glucosidase activity (U*·*L^−1^)	Protein concentration (mg*·*L^−1^)
1	4.0	0.6	0.6	0.5	123.5 ± 33.1	2984.6 ± 39.1	284.8 ± 4.7	70.9 ± 3.3
2	8.0	0.6	0.6	0.5	128.5 ± 35.9	1749.6 ± 35.4	222.2 ± 3.1	57.9 ± 2.0
3	4.0	1.8	0.6	0.5	177.0 ± 23.7	2100.6 ± 31.4	363.8 ± 2.9	93.7 ± 2.4
4	8.0	1.8	0.6	0.5	89.7 ± 9.3	2356.3 ± 10.3	243.6 ± 10.3	72.1 ± 6.5
5	4.0	0.6	1.8	0.5	84.4 ± 6.5	496.3 ± 7.1	47.7 ± 2.9	81.7 ± 4.7
6	8.0	0.6	1.8	0.5	135.2 ± 15.4	3985.6 ± 28.9	805.0 ± 5.6	119.3 ± 3.6
7	4.0	1.8	1.8	0.5	105.5 ± 14.4	2281.1 ± 29.2	233.8 ± 12.4	63.3 ± 4.8
8	8.0	1.8	1.8	0.5	68.4 ± 17.1	726.9 ± 7.1	176.1 ± 7.3	35.8 ± 3.1
9	4.0	0.6	0.6	1.5	250.4 ± 2.1	5509.6 ± 33.8	915.3 ± 3.8	143.3 ± 1.2
10	8.0	0.6	0.6	1.5	226.6 ± 2.4	4943.1 ± 27.4	732.6 ± 8.7	150.5 ± 9.1
11	4.0	1.8	0.6	1.5	178.9 ± 3.3	2772.4 ± 9.2	439.5 ± 2.2	101.5 ± 3.2
12	8.0	1.8	0.6	1.5	140.7 ± 9.8	5344.2 ± 10.8	816.5 ± 5.7	176.4 ± 8.1
13	4.0	0.6	1.8	1.5	2.5 ± 1.3	451.2 ± 4.1	13.2 ± 2.9	59.9 ± 2.2
14	8.0	0.6	1.8	1.5	24.3 ± 2.2	588.2 ± 11.5	139.9 ± 2.8	31.3 ± 2.4
15	4.0	1.8	1.8	1.5	74.0 ± 1.8	56.8 ± 2.1	34.6 ± 4.9	45.6 ± 0.4
16	8.0	1.8	1.8	1.5	66.6 ± 3.3	207.2 ± 15.3	60.9 ± 2.9	43.2 ± 2.4
**17** (CP)	**6.0**	**1.2**	**1.2**	**1.0**	**158.6 **± 4.1	**4908.0 **± 23.2	**1345.8 **± 3.5	**110.9 **± 0.3
**18** (CP)	**6.0**	**1.2**	**1.2**	**1.0**	**170.3 **± 2.4	**5153.7 **± 27.9	**1320.3 **± 4.1	**144.8 **± 4.8
**19** (CP)	**6.0**	**1.2**	**1.2**	**1.0**	**172.7 **± 1.1	**5660.0 **± 32.6	**1326.0 **± 5.9	**153.5 **± 3.9
**20** (CP)	**6.0**	**1.2**	**1.2**	**1.0**	**178.2 **± 1.8	**4481.9 **± 28.6	**1234.7 **± 13.9	**77.6 **± 1.5
**21** (CP)	**6.0**	**1.2**	**1.2**	**1.0**	**161.7 **± 7.7	**5190.5 **± 23.5	**1128.5 **± 11.2	**128.1 **± 1.3

^a^ *A*, *B*, *C*, *D*: see Table 1. CP: center point.

**Table 5:** Cellulase activities and protein concentration for the 3^2^ FFD using an instrumented bioreactor.

Run	*E* ^ a^	*F* ^ a^	FPase activity (U*·*L^−1^)	Endoglucanase activity (U*·*L^−1^)	*β*-Glucosidase activity (U*·*L^−1^)	Protein concentration (mg*·*L^−1^)
1	100	0.2	125.6 ± 6.8	3184.5 ± 18.2	905.9 ± 7.4	52.5 ± 0.7
2	100	0.6	78.0 ± 3.4	2584.9 ± 14.3	771.8 ± 6.0	47.3 ± 1.8
3	100	1.0	163.6 ± 12.8	770.5 ± 2.6	185.0 ± 3.5	91.3 ± 2.9
4	200	0.2	190.8 ± 5.3	3706.6 ± 17.1	984.9 ± 8.1	48.8 ± 2.2
**5 (CP)**	**200**	**0.6**	**444.8 **± 8.1	**9579.5 **± 22.2	**2395.2 **± 18.3	**179.2 **± 3.2
6	200	1.0	558.6 ± 13.2	5622.6 ± 27.4	1613.3 ± 19.5	209.4 ± 1.0
7	300	0.2	252.7 ± 3.7	4661.0 ± 15.5	1149.0 ± 13.5	104.7 ± 2.8
8	300	0.6	338.2 ± 4.9	9804.4 ± 15.3	2860.5 ± 3.5	105.5 ± 2.3
9	300	1.0	224.4 ± 9.1	4535.9 ± 13.8	1173.7 ± 6.5	125.3 ± 5.5
**10 (CP)**	**200**	**0.6**	**449.5 **± 3.9	**9784.3 **± 27.4	**2451.0 **± 15.2	**187.5 **± 1.9
**11 (CP)**	**200**	**0.6**	**431.2 **± 5.1	**9396.8 **± 27.1	**2088.7 **± 13.9	**176.3 **± 1.5

^a^ *E*, *F*: see Table 2. CP: center point.

**Table 6:** Experimental validation of optimal conditions for cellulase production, predicted using the multivariate desirability function.

	Agitation (rpm)	Aeration (vvm)	FPase activity (U*·*L^−1^)	Endoglucanase activity (U*·*L^−1^)	*β*-Glucosidase activity (U*·*L^−1^)	Protein concentration (mg*·*L^−1^)
Predicted values						
FPase activity (U*·*L^−1^)	215	0.9	460	—	—	—
Endoglucanase activity (U*·*L^−1^)	245	0.6	—	9626	—	—
*β*-Glucosidase activity (U*·*L^−1^)	260	0.6	—	—	2467	—
Protein concentration (mg*·*L^−1^)	214	1.0	—	—	—	189
Desirability	227.5	0.68	446	9414	2386	174

Observed values						
Bioreactor (120 h)	220	0.6	508 ± 12.1	9204 ± 142.2	2395 ± 24.8	245 ± 15.9
